# Influence of *Strongyloides stercoralis* Coinfection on the Presentation, Pathogenesis, and Outcome of Tuberculous Meningitis

**DOI:** 10.1093/infdis/jiaa672

**Published:** 2020-10-26

**Authors:** Joseph Donovan, Trinh Thi Bich Tram, Nguyen Hoan Phu, Nguyen Thi Thu Hiep, Vu Thi Thu Van, Dang Thi Hong Mui, Nguyen Thi Han Ny, Ho Dang Trung Nghia, Nguyen Ho Hong Hanh, Le Van Tan, Nguyen Thuy Thuong Thuong, Guy E Thwaites

**Affiliations:** 1 Oxford University Clinical Research Unit, Centre for Tropical Medicine, Ho Chi Minh City, Vietnam; 2 Centre for Tropical Medicine and Global Health, Nuffield Department of Medicine, University of Oxford, Oxford, United Kingdom; 3 School of Medicine, Vietnam National University of Ho Chi Minh City, Ho Chi Minh City, Vietnam; 4 Hospital for Tropical Diseases, Ho Chi Minh City, Vietnam; 5 Pham Ngoc Thach University of Medicine, Ho Chi Minh City, Vietnam

**Keywords:** *Strongyloides stercoralis*, tuberculous meningitis, immunomodulation, cytokines, inflammation, outcome

## Abstract

**Background:**

Helminth infections may modulate the inflammatory response to *Mycobacterium tuberculosis* and influence disease presentation and outcome. *Strongyloides stercoralis* is common among populations with high tuberculosis prevalence. Our aim was to determine whether *S. stercoralis* coinfection influenced clinical presentation, cerebrospinal fluid (CSF) inflammation, and outcome from tuberculous meningitis (TBM).

**Methods:**

From June 2017 to December 2019, 668 Vietnamese adults with TBM, enrolled in the ACT HIV or LAST ACT trials (NCT03092817 and NCT03100786), underwent pretreatment *S. stercoralis* testing by serology, stool microscopy, and/or stool polymerase chain reaction. Comparisons of pretreatment TBM severity, CSF inflammation (including cytokines), and 3-month clinical end points were performed in groups with or without active *S. stercoralis* infection.

**Results:**

Overall, 9.4% participants (63 of 668) tested positive for *S. stercoralis*. Active *S. stercoralis* infection was significantly associated with reduced pretreatment CSF neutrophil counts (median [interquartile range], 3/μL [0–25/μL] vs 14 /μL [1–83/μL]; *P = *.04), and with reduced CSF interferon ɣ, interleukin 2, and tumor necrosis factor α concentrations (11.4 vs 56.0 pg/mL [*P = *.01], 33.1 vs 54.5 pg/mL [*P = *.03], and 4.5 vs 11.9 pg/mL [*P = *.02], respectively), compared with uninfected participants. Neurological complications by 3 months were significantly reduced in participants with active *S. stercoralis* infection compared with uninfected participants (3.8% [1 of 26] vs 30.0% [33 of 110], respectively; *P = *.01).

**Conclusions:**

*S. stercoralis* coinfection may modulate the intracerebral inflammatory response to *M. tuberculosis* and improve TBM clinical outcomes.

## Teaser

In Vietnamese adults with tuberculous meningitis (TBM), active *Strongyloides stercoralis* coinfection was significantly associated with reduced intracerebral inflammation and reduced neurological complications by 3 months, compared with *S. stercoralis*–uninfected participants. *S. stercoralis* coinfection may modulate neuroinflammatory response, and improve outcome, in TBM.

The soil-transmitted helminth *Strongyloides stercoralis* causes strongyloidiasis, a neglected chronic parasitic disease of humans. Found throughout tropical and subtropical regions of the world, *S. stercoralis* infects an estimated 30–100 million individuals globally [1]. The geographic distribution of *S. stercoralis* overlaps with that of tuberculosis. Tuberculous meningitis (TBM) is the most severe form of tuberculosis, resulting in death in almost half of all cases, despite effective antituberculosis chemotherapy [2–5]. TBM is characterized by intracerebral inflammation, which can lead to fatal complications.

Helminth coinfection appears to modulate the host immune response to *Mycobacterium tuberculosis* infection and may increase susceptibility to developing disease (tuberculosis) and worsen its severity [6]. Helminth infections typically induce a T-helper (Th) 2 immune response, with an immunoglobulin E antibody class switch, production and activation of eosinophils, mast cell degranulation [7], and marked elevation of interleukin 4, 5, and 13 (IL-4, IL-5, and IL-13) [6]. Th2 responses appear to be cross-inhibitory with the proinflammatory Th1 immune responses associated with tuberculosis [8, 9]. In a case-control study of 40 individuals with pulmonary tuberculosis, significantly lower blood interferon (IFN) ɣ levels and a nonsignificant trend toward more severe disease were found in helminth-coinfected individuals compared with helminth uninfected controls [10]. A study of proinflammatory cytokines in patients with pulmonary tuberculosis (n = 88; 42 of 88 coinfected with *S. stercoralis*) and latent tuberculosis (n = 88; 44 of 88 coinfected with *S. stercoralis*) found significantly lower plasma tumor necrosis factor (TNF) α, IFN-ɣ, and interleukin 2 (IL-2) in *S. stercoralis*–coinfected individuals, compared with a tuberculosis-only control group [11]. In addition plasma concentrations of anti-inflammatory cytokines interleukin 10 (IL-10), IL-4, IL-5, and IL-13 were significantly elevated in individuals with latent tuberculosis and *S. stercoralis* compared with those with latent tuberculosis alone.

The intracerebral inflammation of TBM is poorly understood. A Th1 immune response is typical, with phagocytosis, intracellular killing of microbes [7], and elevated cerebrospinal fluid (CSF) concentrations of proinflammatory cytokines [12–14], such as TNF-α and IFN-γ. However, previous studies have shown substantial heterogeneity in the response, with poor outcomes associated with both excessive and attenuated inflammatory responses [15–17]. The determinants of this heterogeneity are uncertain; host genetic variation in leukotriene A4 hydrolase (*LTA4H*) may play a role in some populations [18, 19], but other determinants are likely. Here, we examine the hypothesis that helminth coinfection modulates the intracerebral inflammatory response to *M. tuberculosis* and thus influences the clinical presentation and outcomes of TBM.

## METHODS

### Participants

We performed a prospective study in Vietnamese adults with TBM to evaluate the frequency and effect of *S. stercoralis* coinfection on presenting clinical phenotype, CSF inflammatory parameters, CSF cytokine concentrations, and clinical end points. Participants were enrolled from 2 ongoing randomized placebo-controlled phase III trials of adjunctive corticosteroid therapy for human immunodeficiency virus (HIV)–coinfected and HIV-uninfected adults with TBM (ACT HIV [NCT03092817 [20]] and LAST ACT [NCT03100786 [21]]).

Participants were ≥18 years old, with a diagnosis of TBM based on consistent clinical and CSF findings, with or without HIV coinfection, and admitted to the Hospital for Tropical Diseases or Pham Ngoc Thach Hospital for Tuberculosis and Lung Disease, both in Ho Chi Minh City, Vietnam. Patients were excluded if an additional brain infection to TBM was suspected, if they received >6 consecutive days of antituberculosis chemotherapy or systemic corticosteroids, or if corticosteroids were mandatory or contraindicated.

Written informed consent was obtained from all participants or from a relative if the participant was incapacitated. Ethical approvals for ACT HIV and LAST ACT were obtained from the Oxford Tropical Research Ethics Committee (nos. 36-16 and 52-16, respectively), the ethical committees of the Hospital for Tropical Diseases (nos. 14/HDDD and 37/HDDD, respectively) and Pham Ngoc Thach Hospital for Tuberculosis and Lung Disease (nos. 1033/HDDD-PNT and 460/HDDD-PNT, respectively), and from the Vietnam Ministry of Health (nos. 108/CN-BDGDD and 151/CN-BDGDD, respectively).

### Clinical Data

Demographic data (age, sex), baseline Modified Research Council (MRC) TBM severity grade [22], and HIV status were recorded. Study participants were followed up for 3 months. Death and neurological complications by 3 months were recorded. Neurological complications were defined as a fall in Glasgow coma score of ≥2 points for ≥48 hours, a focal neurological sign, seizure, cerebellar signs, coma, or cerebral herniation.

### Laboratory Testing

All participants enrolled in this study underwent ≥1 test for *S. stercoralis* infection. *S. stercoralis s*erology (NovaTec Immunodiagnostica) was performed in participants at baseline (date of signing informed consent). Routine wet preparation stool microscopy was performed in participants within 7 days of baseline. Stool *S. stercoralis* PCR testing was performed in a subgroup of participants (those testing positive for *S. stercoralis* at serology or stool microscopy [allowing comparison of diagnostic tests] and in consecutively enrolled participants until a total of 200 PCR tests had been performed). Blood eosinophil count was measured at baseline in all participants.

At least 6 mL of lumbar CSF was sampled (if available) at baseline in all participants. CSF processing and testing followed procedures described elsewhere [23]. CSF supernatant was removed and stored at −80^0^C for future CSF cytokine testing. Cytokines were selected for CSF analysis based on previous CSF cytokine studies in TBM, as described elsewhere, cytokines predicted to be affected by *S. stercoralis* coinfection, [11] and availability of testing kits; CSF TNF-α, IFN-γ, interleukin 6 and 12p7 (IL-6 and IL-12p70), IL-1β, IL-2, IL-4, IL-5, IL-10, and IL-13 were measured. CSF cytokine testing was performed using magnetic microbead immunoassay (R&D Systems), following the manufacturer’s instructions [24]. Cytokine concentrations were measured using a Luminex 200 instrument (Luminex). Luminex 200 xPONENT software (version 3.1.971.0) was used for analysis.

### Treatment

All participants received antituberculosis chemotherapy following national guidelines. Rifampicin, isoniazid, pyrazinamide, and ethambutol were given for at least the first 2 months, if drug resistance was not suspected or proved. Pyrazinamide was stopped after 2 months. At least 12 months of antituberculosis chemotherapy was received in total. Antituberculosis chemotherapy regimens are further described in the Supplementary Material. Participants with a positive result of stool microscopy or a PCR test for *S. stercoralis* received oral ivermectin (200 µg/kg/d for 10–14 days), with repeated stool microscopy required to demonstrate absence of *S. stercoralis* larvae. Participants with positive results of *S. stercoralis* serology were treated on a case-by-case basis. In addition, all participants were randomized to dexamethasone or placebo (termed “study drug”), a double-blinded allocation following 1:1 randomization (except *LTA4H* TT-genotype HIV-uninfected participants from the LAST ACT trial [approximately 7% of total participants] who all received open-label dexamethasone). The study drug was administered over 6–8 weeks, following a tapering course, with weekly reductions (Supplementary Table 1). The ACT HIV and LAST ACT trials are ongoing, and treatment allocations remain blinded. Permission to publish these study data was obtained by the respective trial steering committees.

### Statistical Analysis

Primary analysis populations were selected based on clinical categories of *S. stercoralis* infection. The *S. stercoralis* “uninfected” group consisted of participants tested with *S. stercoralis* serology, stool microscopy, and stool PCR, with all results negative. This approach gave the highest certainty of a *S. stercoralis*–uninfected status. A “past infection” group consisted of participants with positive results of *S. stercoralis* serology, with no positive stool result (but with stool microscopy and/or stool PCR performed). An “active infection” group consisted of participants with positive *S. stercoralis* stool microscopy or stool PCR results, regardless of other testing.

Secondary analyses were performed on 2 additional subpopulations; participants who had serology performed, and those who had both serology and stool microscopy performed (divided into groups A–C) (Supplementary Tables 2 and 3). Secondary analyses compared baseline TBM severity, CSF inflammatory parameters, and clinical end points between participants with or without positive *S. stercoralis* test results, for each subpopulation. CSF cytokine analysis was performed only for primary analysis populations.

Where CSF cytokine concentrations were undetected, either the lowest limit of extrapolation divided by 2, or the lowest limit of detection divided by 2, was used, whichever was lowest. CSF cytokine testing was performed across two 96-well plates, and value extrapolation was plate specific. Rarely, where cytokine concentrations were too high for quantification, the upper limit of detection multiplied by 2 was used; samples and testing kits were unavailable for sample dilution and repeated testing. Log_2_ calculations of CSF cytokine concentration were performed. Given the unknown magnitude of *S. stercoralis* immunomodulation of CSF cytokine concentrations, sample size calculation was not possible. The number of CSF samples undergoing CSF cytokine analysis was based on availability of sample and testing kits and was therefore exploratory. Clinical data proportions were compared using χ2 tests, and CSF cytokine concentrations using Wilcoxon rank sum tests. Multivariate analysis (with odds ratios and 95% confidence intervals) was performed to evaluate whether age, MRC TBM grade, HIV coinfection, and active *S. stercoralis* infection predicted neurological complications by 3 months. Data were analyzed using R software (version 3.6).

## RESULTS

### Study Population

From June 2017 to December 2019 inclusive, 668 participants with TBM underwent baseline testing for *S. stercoralis* coinfection, with serology, stool microscopy, and/or stool PCR. The median age of the study population (interquartile range [IQR]) was 39 (31–50) years, and 67.5% of the participants (451 of 668) were male. The MRC TBM severity grade [22, 25] in the study population was grade 1 in 45.1% of participants (n = 301), grade 2 in 43.3% (n = 289), and grade 3 in 11.7% (n = 78). Of the study participants, 43.4% (n = 290) had a diagnosis of definite TBM; 38.3% (n = 256), probable TBM; and 16.0% (n = 107), possible TBM; 44.6% of participants (298 of 668) were HIV coinfected.

### *S. stercoralis* Testing

**Figure 1.**
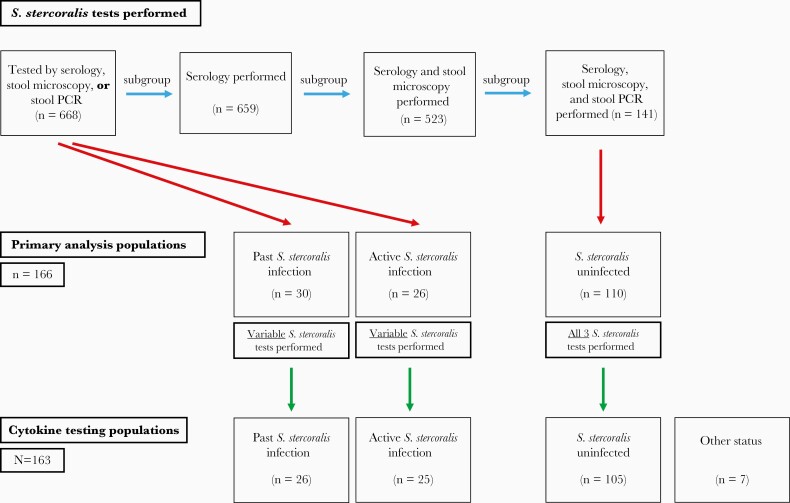
*Strongyloides stercoralis* testing populations. A total of 668 participants underwent ≥1 *S. stercoralis* test, serology in 659, serology and stool microscopy in 523, and serology, stool microscopy, and stool polymerase chain reaction (PCR) in 141. Following the light gray arrows, each group is a subgroup of the previous group. Dark gray arrows show how primary analysis populations were developed. All *S. stercoralis*–uninfected participants had serology, stool microscopy, and stool PCR performed. Past *S. stercoralis* infection and active *S. stercoralis* infection groups were selected independently of the number of *S. stercoralis* tests performed; therefore, these are taken from the population in which ≥1 *S. stercoralis* test was performed (N = 668). Black arrows show how cytokine testing populations were formed. From a total of 173 patients initially eligible for cytokine testing, 10 samples were omitted; 4 were excluded from testing when no stored cerebrospinal fluid (CSF) sample was available, 5 were not tested because *S. stercoralis* tests returned a positive result after cytokine testing had been arranged and set up, and 1 sample result was lost owing to a computer error during cytokine analysis. Therefore, 163 CSF samples underwent cytokine testing, of which 156 fit into primary analysis population definitions (uninfected, past infection, or active infection). For the uninfected group, all 3 testing methods were used, all with negative results. For the past infection group, results of *S. stercoralis* serology were positive with no positive stool testing results (but with stool microscopy and/or stool PCR performed). In the active infection group, results of stool microscopy or stool PCR were positive for *S. stercoralis*, regardless of other testing performed. The “Other status” group includes participants who underwent cytokine testing but did not meet criteria for any of the 3 primary analysis population groups.

**Figure 2.**
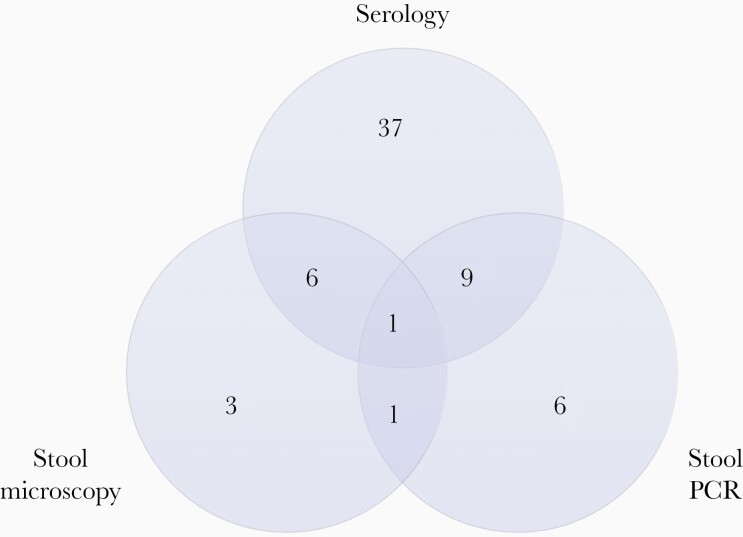
Venn diagram of 81 *Strongyloides stercoralis* tests with positive results, including serology (n = 53), stool microscopy (n = 11), and stool polymerase chain reaction (PCR) (n = 17). The tests were performed in 63 participants testing positive for *S. stercoralis* with serology, stool microscopy, and/or stool PCR; these participants include the past infection (n = 30) and active infection (n = 26) primary analysis populations, as well as 7 participants with a positive *S. stercoralis* test result not meeting the criteria for those populations.

The total numbers of *S. stercoralis* tests performed are shown in Figure 1. Overall, 9.4% of participants (63 of 668) tested positive by *S. stercoralis* serology (n = 53), stool microscopy (n = 11), and/or stool PCR (n = 17) (Figure 2). All 3 diagnostic tests were performed in 141 of 668 participants (21.1%). A positive *S. stercoralis* diagnosis was made by means of stool microscopy alone in 3 participants and stool PCR alone in 6. The median age (IQR) of participants testing positive for *S. stercoralis* by any method was 49 (37–59) years, versus 40 (32–51) years in those who tested negative for *S. stercoralis* with serology, stool microscopy, and stool PCR. Of *S. stercoralis*–positive participants 55 of 63 (87.3%) were male, compared with 75 of 110 (68.2%) for *S. stercoralis*–negative participants. HIV coinfection was present in 16 of 63 *S. stercoralis*–positive participants (25.4%) and in 37 of 110 *S. stercoralis*–negative participants (33.6%).

### Influence of *S. stercoralis* Infection on TBM Presentation and Routine CSF Parameters

A comparison of baseline TBM severity and routine CSF parameters between primary analysis populations is shown in Table 1 [26]. Baseline blood eosinophil counts were significantly elevated in active *S. stercoralis* infection compared with *S. stercoralis*–uninfected participants (median [IQR], 0.10 [0–0.38] ×10^9^/L vs [0–0.10] 0 ×10^9^/L), respectively (*P = *.02). The median (IQR) CSF neutrophil count and neutrophil percentage were reduced in active *S. stercoralis* infection compared with uninfected participants (neutrophil count, 3/μL [0–25/μL] vs 14/μL (1–83/μL); neutrophil percentage, 5% [0%–14%] vs 10% [5%–27%]; both *P = *.04). In addition, in participants with active *S. stercoralis* infection, compared with uninfected participants, trends were seen toward reduced grade 3 disease (3.8% [1 of 26 participants] vs 19.1% [21 of 110], respectively), reduced total CSF white blood cell (WBC) count (median [IQR], 70/μL [7–168/μL] vs 123/μL [29–297/μL]), reduced CSF protein (median [IQR], 0.94 [0.60–1.84] vs 1.45 [0.95–2.18] g/L), and elevated CSF/blood glucose ratio (median [IQR], 0.45 [0.31–0.59] vs 0.38 [0.26–0.52]).

**Table 1.** Baseline Tuberculous Meningitis Severity and Cerebrospinal Fluid Inflammatory Parameters by Primary Analysis Population

	Analysis Population by *Strongyloides stercoralis* Infection Status^a^				
Parameter	Uninfected (n = 110)	Past Infection (n = 30)	*P* Value	Active Infection (n = 26)	*P* Value
HIV status, no. (%)					
Infected	37 (33.6)	4 (13.3)	.05	9 (34.6)	>.99
Uninfected	73 (66.4)	26 (86.7)		17 (65.4)	
Final diagnosis, no. (%)^b^					
Definite TBM	58 (52.7)	9 (30.0)	Reference^c^	5 (19.2)	Reference^c^
Probable TBM	38 (34.5)	14 (46.7)	.11	16 (61.5)	.01
Possible TBM	13 (11.8)	6 (20.0)	.13	5 (19.2)	.06
MRC TBM grade, no. (%)^d^					
1	44 (40.0)	16 (53.3)	Reference^c^	13 (50.0)	Reference^c^
2	45 (40.9)	12 (40.0)	.62	12 (46.2)	>.99
3	21 (19.1)	2 (6.7)	.14	1 (3.8)	.11
Laboratory values, median (IQR)					
Baseline eosinophil count,10^9^ cells/L	0 (0–0.10)	0.2 (0.08–0.20)	<.001	0.1 (0–0.38)	.02
CSF WBC count, cells/μL	123 (29–297)	74 (9–254)	.20	70 (7–168)	.13
CSF neutrophil count, cells/μL	14 (1–83)	6 (0–36)	.25	3 (0–25)	.04
CSF neutrophils, %	10 (5–27)	11 (0–15)	.20	5 (0–14)	.04
CSF/blood glucose ratio	0.38 (0.26–0.52)	0.43 (0.33–0.52)	.34	0.45 (0.31–0.59)	.16
CSF protein, g/L	1.45 (0.95–2.18)	1.39 (1.13–1.94)	.69	0.94 (0.60–1.84)	.08
GeneXpert MTB/RIF assay result, no. (%)					
Positive	31 (28.2)	6 (20.0)	.50	1 (3.8)	.03
Negative	68 (61.8)	21 (70.0)		24 (92.3)	

Abbreviations: CSF, cerebrospinal fluid; HIV, human immunodeficiency virus; IQR, interquartile range; MRC, Modified Research Council; TBM, tuberculous meningitis; WBC, white blood cell.

^a^In the uninfected group, all 3 testing methods were used, and all results were negative. In the past infection group, results of *S. stercoralis* serology were positive, but results of stool testing were negative, after performance of stool microscopy and/or stool polymerase chain reaction (PCR). In the active infection group, results of stool microscopy or stool PCR were positive for *S. stercoralis,* regardless of other testing performed. *P* values represent comparisons between the *S. stercoralis*–uninfected group and either the uninfected or the active infection group, with χ2 and Wilcoxon rank sum tests used to compare categorical and continuous data, respectively.

^b^For final diagnosis categories, *P* values represent comparison between definite TBM and either probable and possible TBM, with these categories defined according to the published uniform case definitions for TBM [26]. One participant in the uninfected group did not have CSF parameters available and could not be classified as definite, probable, or possible TBM. One in the past infection group scored <6 points for the TBM diagnostic score [26]. Both cases were considered to be TBM by the treating clinician and were treated as such.

^c^Reference standard (final diagnosis or grade against which comparison was made).

^d^For MRC TBM grades, *P* values represent comparison between grade 1 TBM and either grade 2 or grade 3 TBM.

There was a reduced proportion of definite TBM in the active *S. stercoralis* group versus the *S. stercoralis*–uninfected group (19.2% [5 of 26 participants] vs 52.7% [58 of 110]; *P* = .01 for definite vs probable TBM). In addition, there was reduced positivity with the GeneXpert MTB/RIF assay in the active *S. stercoralis* compared with the *S. stercoralis*–uninfected group (3.8% [1 of 26 participants] vs 28.2% [31 of 110], respectively; *P = *.03). CSF/blood glucose ratios were significantly higher, and CSF protein levels significantly lower, in the active *S. stercoralis* versus the *S. stercoralis*–uninfected group, in a HIV-coinfected subgroup (Supplementary Table 4).

Supplementary Tables 2 and 3 show results of baseline TBM severity and CSF inflammatory parameter analyses in subpopulations of participants in whom serology or both serology and stool microscopy were performed. In participants with *S. stercoralis* serology performed, HIV coinfection was less common in those with positive than in those with negative serological results (20.8% [11 of 53 participants] vs 46.2% [280 of 606], respectively; *P* = .001).

### Baseline CSF Cytokine Concentrations in *S. stercoralis* Coinfection

We hypothesized that in participants with active *S. stercoralis* infection, CSF concentrations of the proinflammatory cytokines IFN-ɣ, IL-2, and TNF-α would be reduced and CSF concentrations of the regulatory cytokines IL-4, IL-5, IL-10, and IL-13 would be increased, compared with *S. stercoralis*–uninfected participants. These cytokines, in addition to IL-1β, IL-6, and IL-12p70 (as exploratory analyses), were measured in CSF and compared between primary analysis populations.

**Figure 3.**
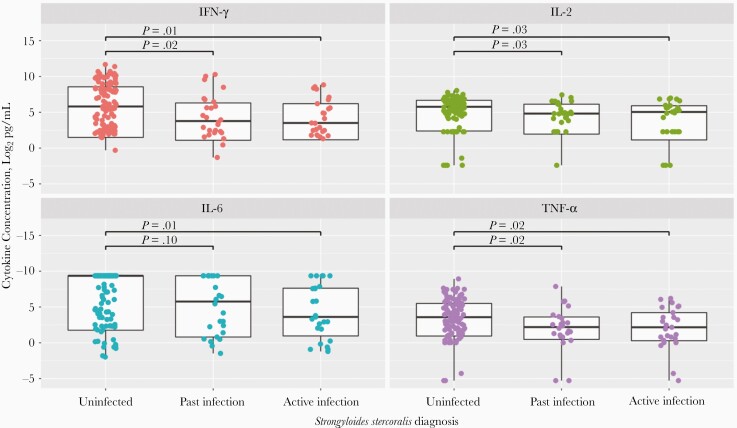
Log_2_ cerebrospinal fluid interferon (IFN) ɣ, interleukin 2 (IL-2), interleukin 6 (IL-6), and tumor necrosis factor (TNF) α concentrations in participants uninfected with *Strongyloides stercoralis*, with past *S. stercoralis* infection, or with active infection. The log_2_ cytokine concentrations 15, 10, 5, 0, and −5 correspond to the following measured cytokine concentrations: 32 768, 1024, 32, 1, and 0.03 pg/mL, respectively. For each individual box plot, the central horizontal bar represents the median value, and the box contains data between the third and first quartiles (upper and lower ends of box, respectively); vertical lines above and below each box extend to the most extreme data point within 1.5 times the height of the box; and dots represent individual data points. For the uninfected group, all 3 testing methods were used, all with negative results. For the past infection group, results of *S. stercoralis* serology were positive with no positive stool testing results (but with stool microscopy and/or stool polymerase chain reaction [PCR] performed). In the active infection group, results of stool microscopy or stool PCR were positive for *S. stercoralis*, regardless of other testing performed. Statistical comparisons of cytokine concentrations were performed using Wilcoxon rank sum tests.

CSF cytokine testing populations are shown in Figure 1. CSF concentrations of proinflammatory cytokines were significantly reduced in participants with active *S. stercoralis* infection (n = 25), compared with uninfected participants (n = 105) (IFN-ɣ, 11.4 vs 56.0 pg/mL [*P = *.01]; IL-2, 33.1 vs 54.5 pg/mL [*P = *.03]; TNF-α, 4.5 vs 11.9 pg/mL [*P = *.02]; IL-6, 12.2 vs 655.6 pg/mL [*P = *.01]) (Figure 3). In addition, CSF concentrations of IFN-ɣ, TNF-α, IL-2, but not IL-6, were significantly reduced in participants with past *S. stercoralis* infection (n = 26), compared with uninfected participants (IFN-ɣ, 13.8 vs 56.0 pg/mL [*P = *.02]; IL-2, 28.3 vs 54.5 pg/mL [*P = *.03]; TNF-α, 4.6 vs 11.9 pg/mL [*P = *.02]; IL-6, 55.4 vs 655.6, pg/mL [*P = *.10]).

**Figure 4.**
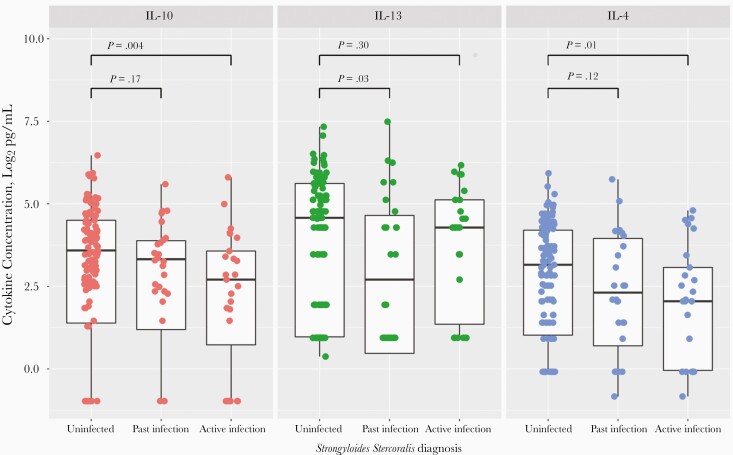
Log_2_ cerebrospinal fluid interleukin 10, 13, and 4 (IL-10, IL-13, and IL-4) concentrations in participants uninfected with *Strongyloides stercoralis*, with past *S. stercoralis* infection, or with active infection. For each individual box plot, the central horizontal bar represents the median value, and the box contains data between the third and first quartiles (upper and lower ends of box, respectively); vertical lines above and below each box extend to the most extreme data point within 1.5 times the height of the box; and dots represent individual data points. For the uninfected group, all 3 testing methods were used, all with negative results. For the past infection group, results of *S. stercoralis* serology were positive with no positive stool testing results (but with stool microscopy and/or stool polymerase chain reaction [PCR] performed). In the active infection group, results of stool microscopy or stool PCR were positive for *S. stercoralis*, regardless of other testing performed. Statistical comparison of cytokine concentrations was performed using Wilcoxon rank sum tests.

Contrary to our hypothesis, CSF concentrations of IL-10 and IL-4 were significantly reduced in active *S. stercoralis* infection compared with *S. stercoralis*–uninfected participants (IL-10, 6.5 vs 12.0 pg/mL [*P = *.004]; IL-4, 4.1 vs 8.9 pg/mL [*P = *.01]) (Figure 4). Seventy percent of participants had undetectable IL-5 in their CSF samples. In participants with past *S. stercoralis* infection, compared with uninfected participants, CSF concentrations of IL-13 were reduced (7.5 vs 23.9 pg/mL; *P = *.03) and CSF concentrations of IL-5 were increased (0.37 vs 0.37 pg/mL; *P = *.02). Median cytokine concentrations for *S. stercoralis*–uninfected, past infection, and active infection groups, together with ratio of change and statistical comparison between groups, are shown in Supplementary Table 5. IL-6 concentrations showed the greatest ratio of reduction, approximately a 54-fold reduction in active *S. stercoralis* infection compared with uninfected participants. CSF concentrations of TNF-α, IFN-γ, IL-1β, IL-2, IL-4, IL-6, and IL-10 were significantly reduced in participants with active *S. stercoralis* infection, compared with uninfected participants, in an HIV-coinfected subgroup; however, these significant differences were not seen in an HIV-uninfected subgroup (Supplementary Table 6).

### *S. stercoralis* Coinfection and Outcome from TBM

Table 2 compares neurological complications by 3 months, and death by 3 months, between primary analysis populations. Neurological complications by 3 months were significantly reduced in participants with active *S. stercoralis* infection (3.8% [1 of 26]), compared with uninfected participants (30.0% [33 of 110]; *P = *.01). Neurological complications are listed in Supplementary Table 7. A fall in Glasgow coma score ≥2 points for ≥48 hours was the most common neurological complication recorded, accounting for 80% (4 of 5) neurological complications in past *S. stercoralis* infection, and 78.8% (26 of 33) in *S. stercoralis*–uninfected participants. In a multivariate logistic regression, active *S. stercoralis* infection was significantly and independently associated with reduced neurological events by 3 months (*P* = .01) (Supplementary Table 8). Death by 3 months was not significantly reduced between active *S. stercoralis* infection and uninfected groups (15.4% [4 of 26 participants] vs 28.2% [31 of 110], respectively; *P = *.27). Neurological complications by 3 months remained significantly reduced in participants with active *S. stercoralis* infection versus uninfected participants in HIV uninfected individuals but not in those who were HIV coinfected (Supplementary Table 9).

**Table 2.** Neurological Complications and Death by 3 Months by Primary Analysis Population

	Analysis Population by *Strongyloides stercoralis* Status^a^				
Outcome by 3 mo	Uninfected (n = 110)	Past Infection (n = 30)	*P* Value	Active Infection (n = 26)	*P* Value
Neurological complications, no. (%)					
Yes	33 (30.0 )	5 (16.7)	.22	1 (3.8)	.01
No	77 (70.0)	25 (83.3)		25 (96.2)	
Death, no. (%)					
Yes	31 (28.2)	5 (16.7)	.30	4 (15.4)	.27
No	79 (71.8)	25 (83.3)		22 (84.6)	

^a^In the uninfected group, all 3 testing methods were used, and all results were negative. In the past infection group, results of *S. stercoralis* serology were positive, but results of stool testing were negative, after performance of stool microscopy and/or stool polymerase chain reaction (PCR). In the active infection group, results of stool microscopy or stool PCR were positive for *S. stercoralis,* regardless of other testing performed. *P* values are shown for comparison between the *S. stercoralis*–uninfected group and either the uninfected or the active infection group, with χ2 tests used to compare categorical data.

Additional secondary subpopulation comparisons of neurological complications and death by 3 months, in participants with or without positive *S. stercoralis* results, are shown Supplementary Tables 10 and 11. In participants with serology performed (n = 659), a reduction in deaths at 3 months was suggested in those with positive serological results (15.1% [8 of 53] vs 25.7% [156 of 606], respectively; *P = *.12). For participants in whom both *S. stercoralis* serology and stool microscopy were performed, neither neurological events by 3 months nor death by 3 months differed significantly between participants with positive results of both *S. stercoralis* serology and stool microscopy, positive results of *S. stercoralis* serology but negative results of stool microscopy, or negative results of *S. stercoralis* serology (Supplementary Table 11).

## DISCUSSION

*S. stercoralis* is a neglected tropical infection with a huge global disease burden. The ability of helminths to modulate host immunity is well recognized; however, immunomodulation of the intracerebral inflammatory responses associated with TBM has not previously been described to our knowledge. In our study of 668 Vietnamese adults with TBM, active *S. stercoralis* infection was associated with reduced intracerebral inflammation and reduced neurological events by 3 months, compared with *S. stercoralis*–uninfected participants. This association was strongest in HIV-coinfected participants.

In TBM, intracerebral inflammation manifests as abnormal routine CSF parameters (elevated total WBC counts, neutrophil counts, and protein levels and reduced glucose levels) and elevated proinflammatory CSF cytokine concentrations [12, 13, 27]. In our study, active *S. stercoralis* infection was associated with significant reductions in absolute CSF neutrophil count and neutrophil proportion and nonsignificant reductions in CSF total WBC, CSF protein, and an increase in CSF/blood glucose ratio. The reduced inflammatory CSF profile in active *S. stercoralis* infection was consistent with the trend toward reduced grade 3 TBM disease in this group, compared with *S. stercoralis*–uninfected participants.

Significantly reduced “definite” TBM cases (which require microbiological confirmation of *M. tuberculosis*) and positive GeneXpert MTB/RIF assay results in active *S. stercoralis* infection suggest reduced mycobacterial burden in these participants. We speculate that these findings may reflect better host immunological control of TBM disease in the context of *S. stercoralis* infection.

The CSF cytokine analysis further supports a model of reduced intracerebral inflammation in TBM in active *S. stercoralis* coinfection. Pretreatment CSF cytokine concentration analysis showed significantly reduced concentrations of the proinflammatory cytokines IFN-γ, IL-2, and TNF-α, in active *S. stercoralis* coinfection. *S. stercoralis* coinfection in TBM was also associated with significantly reduced CSF IL-4 and IL-10, cytokines associated with a Th2 immune response. The suppression of these cytokines does not fit our prior hypothesis, indicating more work to understand the mechanisms of *S. stercoralis* immunomodulation is needed. Previous data in fact show IL-10 levels to be elevated in TBM, decreasing after antituberculosis chemotherapy [12, 13]. Neutrophils highly express IL-4 and IL-10 in *M. tuberculosis* infection [28]; therefore, a reduction in CSF IL-4 and IL-10 concentrations in *S. stercoralis*–coinfected TBM be may mediated through reduced CSF neutrophils. Interestingly, CSF cytokine suppression was greater in HIV-coinfected than in HIV-uninfected participants. HIV coinfection is associated with globally increased CSF cytokines in TBM [19], but why helminth coinfection would control CSF cytokines more in the context of HIV coinfection is unknown and a topic for future research.

Our data showed a significant reduction in neurological complications within 3 months in active *S. stercoralis* infection, compared with *S. stercoralis*–uninfected participants. This finding is consistent with the associations observed between *S. stercoralis* coinfection, reduced bacterial burden, and reduced intracerebral inflammation. In our multivariate analysis, reduced neurological complications could not be explained by differences in age or HIV coinfection between groups. Elevated CSF neutrophil counts have been linked to neurological immune reconstitution inflammatory syndrome in TBM HIV coinfection and to death in HIV-negative TBM disease [16, 29]. Given the known detrimental consequences of excessive intracerebral inflammation to TBM outcomes [19], it is plausible that reduction of neuroinflammation secondary to helminth down-regulation of proinflammatory TBM immune responses reduces neurological complications. Indeed, therapies in severe TBM often attempt to suppress excessive host immune responses. 

This study has limitations. The rate of true *S. stercoralis* coinfection in our study population may be higher than reported, given that not all participants were assessed with serology, stool microscopy and stool PCR. This resulted in the creation of subpopulations for analysis. In addition, the performances of diagnostic tests for *S. stercoralis* are suboptimal. The sensitivity of stool microscopy is low (<30%) [30] owing to intermittent larval shedding. Stool PCR is more sensitive (approximately 65% sensitive) [31], yet some *S. stercoralis* coinfection will still be missed. *S. stercoralis* serological tests are affected by reduced sensitivity in advanced immunosuppression [32, 33] or persistence of serological positivity despite parasite clearance [31]. 

In our subpopulation in which all participants underwent *S. stercoralis* serology, serological results were less likely to be positive in HIV coinfection, possibly reflecting false-negative results in this group. Follow-up in our study was limited to 3 months; longer-term impact on neurological complications or death therefore cannot be assessed. In addition, repeated CSF cytokine analysis, to assess immune responses after *S. stercoralis* eradication, was not performed. Finally, the study drug allocation (dexamethasone or placebo) of the trial participants remains unknown. This will not influence baseline phenotype or pretreatment CSF analyses; all of which represent data or sampling before study drug administration. Given the randomized study drug allocation (1:1), dexamethasone and placebo are expected to be evenly distributed within each individual analysis population.

The strengths of the current study are that it is large and prospective, with careful clinical characterization of TBM and *S. stercoralis* coinfection. It is part of 2 clinical trials with precise treatment protocols and standardized testing and data collection procedures. In this study of TBM, CSF is used for routine parameter and cytokine measurement, allowing a study of inflammation at the site of the disease instead of using blood inflammatory changes to assess intracerebral inflammation.

In conclusion, in our study active *S. stercoralis* coinfection in TBM was associated with reduced intracerebral inflammation and reduced neurological events. Further understanding of these immunomodulatory processes may aid the development of novel host-directed therapies to manage excessive and damaging inflammation of TBM.

## Supplementary Data

Supplementary materials are available at *The Journal of Infectious Diseases* online. Consisting of data provided by the authors to benefit the reader, the posted materials are not copyedited and are the sole responsibility of the authors, so questions or comments should be addressed to the corresponding author.

jiaa672_suppl_Supplementary_Table_1

jiaa672_suppl_Supplementary_Table_2

jiaa672_suppl_Supplementary_Table_3

jiaa672_suppl_Supplementary_Table_4

jiaa672_suppl_Supplementary_Table_5

jiaa672_suppl_Supplementary_Table_6

jiaa672_suppl_Supplementary_Table_7

jiaa672_suppl_Supplementary_Table_8

jiaa672_suppl_Supplementary_Table_9

jiaa672_suppl_Supplementary_Table_10

jiaa672_suppl_Supplementary_Table_11

jiaa672_suppl_Supplementary_Materials_1

## Notes

***Acknowledgments***. The authors acknowledge the molecular biology support of Le Thi Kim Thanh, from the Oxford University Clinical Research Unit. They also acknowledge the laboratory staff at the Hospital for Tropical Diseases in Ho Chi Minh City, the physicians and nurses who cared for the patients at the Hospital for Tropical Diseases and Pham Ngoc Thach Hospital for Tuberculosis and Lung Disease, and the patients who participated.

***Financial support*.** ACT HIV and LAST ACT are supported by the Wellcome Trust (investigator award 110179/Z/15/Z to G. E. T.)

***Potential conflicts of interest***. All authors: No reported conflicts. All authors have submitted the ICMJE Form for Disclosure of Potential Conflicts of Interest. Conflicts that the editors consider relevant to the content of the manuscript have been disclosed.
