# Supplementary material for: Influence of Strongyloides stercoralis Coinfection on the Presentation, Pathogenesis, and Outcome of Tuberculous Meningitis
Source: J Infect Dis. 2020 Oct 26;225(9):1653–62. doi: 10.1093/infdis/jiaa672 (PMC9071290; doi:10.1093/infdis/jiaa672)
Supplement: jiaa672_suppl_Supplementary_Table_1 [file jiaa672_suppl_supplementary_table_1.docx]

**Supplementary table 1: Study drug doses in ACT HIV and LAST ACT after randomisation**[20,21]

|  | **MRC Grade I**  Daily dexamethasone dose/route | **MRC Grades II and III**  Daily dexamethasone dose/route |
| --- | --- | --- |
| Week 1 | 0.3 mg/kg/24 hrs IV | 0.4 mg/kg/24 hrs IV |
| Week 2 | 0.2 mg/kg/24 hrs IV | 0.3 mg/kg/24 hrs IV |
| Week 3 | 0.1 mg/kg/24 hrs IV | 0.2 mg/kg/24 hrs IV |
| Week 4 | 3mg/24 hrs oral | 0.1 mg/kg/24 hrs IV |
| Week 5 | 2mg/24 hrs oral | 4 mg/24 hrs oral |
| Week 6 | 1 mg/24 hrs oral | 3 mg/24 hrs oral |
| Week 7 | Stop | 2 mg/24 hrs oral |
| Week 8 |  | 1 mg/24 hrs oral |

Kg = kilogram; IV = Intravenous; MRC = Medical Research Council
