# Supplementary material for: Influence of Strongyloides stercoralis Coinfection on the Presentation, Pathogenesis, and Outcome of Tuberculous Meningitis
Source: J Infect Dis. 2020 Oct 26;225(9):1653–62. doi: 10.1093/infdis/jiaa672 (PMC9071290; doi:10.1093/infdis/jiaa672)
Supplement: jiaa672_suppl_Supplementary_Table_2 [file jiaa672_suppl_supplementary_table_2.docx]

**Supplementary table 2: A comparison of baseline TBM severity and CSF inflammatory parameters in participants** **who had *S. stercoralis* serology performed**

|  | ***S. stercoralis* testing** | | |
| --- | --- | --- | --- |
|  | **Negative *S. stercoralis* serology** | **Positive *S. stercoralis* serology** | **P value** |
| Patients (No.) | 606 | 53 |  |
| HIV status (No. [%])   - Positive - Negative | 280 (46.2%)  326 (53.8%) | 11 (20.8%)  42 (79.2%) | 0.001 |
| Final diagnosis   - Definite - Probable - Possible | 272 (41.2%)  223 (39.4%)  97 (17.3%) | 16 (30.2%)  26 (49.1%)  10 (18.9%) | Ref  0.05  0.26 |
| MRC TBM Grade (No. [%])   - 1 - 2 - 3 | 267 (44.1%)  266 (43.9%)  73 (12.0%) | 28 (52.8%)  20 (37.7%)  5 (9.4%) | Ref  0.35  0.53 |
| Baseline eosinophil count (10^9^/L)  (Median[IQR]) | 0  (0-0.1) | 0.1  (0-0.3) | 0.002 |
| CSF WBC (cells/mm^3^)  (Median[IQR]) | 75  (6-243) | 70  (7-257) | 0.90 |
| CSF neutrophil count (cells/mm^3^)  (Median[IQR]) | 6  (0-46) | 4  (0-49) | 0.86 |
| CSF neutrophil percentage  (Median[IQR]) | 8  (0-22) | 10  (0-15) | 0.95 |
| CSF/blood glucose ratio  (Median[IQR]) | 0.39  (0.26-0.50) | 0.39  (0.29-0.51) | 0.51 |
| CSF protein (g/L)  (Median[IQR]) | 1.38  (0.73-2.19) | 1.46  (0.91-2.24) | 0.36 |
| Xpert (No. [%])   - Positive - Negative | 161 (26.6%)  410 (67.7%) | 10 (18.9%)  41 (77.4%) | 0.25 |

With final diagnosis, n=2 (negative *S. stercoralis* serology) and n=1 (positive *S. stercoralis* serology) participants scored < 6 points for the TBM diagnostic score.[33]. These were considered to be TBM by the treating clinician and were treated as such. With final diagnosis, P values represent comparison of each of probable and possible TBM, with definite TBM. With MRC TBM Grade P values represent comparison of each of Grade 2 and Grade 3 TBM, with Grade 1 TBM. The chi squared test was used to compare categorical data. The Wilcoxon rank sum test was used to compare continuous data.

Baseline eosinophil counts, CSF WBC, and CSF neutrophil percentage are non-normally distributed and are shown as median (IQR). CSF=Cerebrospinal fluid. HIV=Human immunodeficiency virus. IQR=Interquartile range. MRC=Modified Research Council. TBM=Tuberculous meningitis. WBC=White blood cell. Xpert=GeneXpert MTB/RIF. ‘Ref’ refers to the final diagnosis or grade against which comparison was made.
