# Supplementary material for: Influence of Strongyloides stercoralis Coinfection on the Presentation, Pathogenesis, and Outcome of Tuberculous Meningitis
Source: J Infect Dis. 2020 Oct 26;225(9):1653–62. doi: 10.1093/infdis/jiaa672 (PMC9071290; doi:10.1093/infdis/jiaa672)
Supplement: jiaa672_suppl_Supplementary_Table_4 [file jiaa672_suppl_supplementary_table_4.docx]

**Supplementary table 4: Subgroup analyses of primary *S. stercoralis* analysis populations by HIV co-infection status**

| **HIV uninfected** | | | | | |
| --- | --- | --- | --- | --- | --- |
|  | ***S. stercoralis* testing** | | | | |
|  | **Uninfected** | **Past infection** | | **Active infection** | |
|  |  |  | P value |  | P value |
| Patients (No.) | 73 | 26 |  | 17 |  |
| CSF WBC (cells/mm^3^)  (Median[IQR]) | 140  (49-311) | 85  (34-268) | 0.30 | 72  (52-389) | 0.49 |
| CSF neutrophil count (cells/mm^3^)  (Median[IQR]) | 17  (5-111) | 10  (2-46) | 0.29 | 4  (0-43) | 0.12 |
| CSF/blood glucose ratio  (Median[IQR]) | 0.40  (0.26-0.53) | 0.47  (0.30-0.52) | 0.47 | 0.43  (0.23-0.46) | 0.98 |
| CSF protein (g/L)  (Median[IQR]) | 1.31  (0.90-2.11) | 1.33  (1.13-1.85) | 0.95 | 1.17  (0.60-2.24) | 0.60 |
| **HIV co-infected** | | | | | |
|  | ***S. stercoralis* testing** | | | | |
|  | **Uninfected** | **Past infection** | | **Active infection** | |
|  |  |  | P value |  | P value |
| Patients (No.) | 37 | 4 |  | 9 |  |
| CSF WBC (cells/mm^3^)  (Median[IQR]) | 56  (7-277) | 11  (4-19) | 0.05 | 6  (4-46) | 0.07 |
| CSF neutrophil count (cells/mm^3^)  (Median[IQR]) | 3  (0-52) | 0  (0-1) | 0.13 | 0  (0-7) | 0.16 |
| CSF/blood glucose ratio  (Median[IQR]) | 0.35  (0.26-0.48) | 0.39  (0.37-0.40) | 0.71 | 0.57  (0.45-0.68) | 0.02 |
| CSF protein (g/L)  (Median[IQR]) | 1.75  (1.20-2.19) | 2.02  (1.28-2.53) | 0.77 | 0.60  (0.40-1.17) | 0.03 |

P values are shown for group comparison with *S. stercoralis* uninfected group in each case. Definite, probable and possible TBM labels are allocated based on the published uniform case definition for TBM.[33] The Wilcoxon rank sum test was used to compare continuous data. Uninfected = all 3 testing methods used, and all negative. Past infection = positive *S. stercoralis* serology with no positive stool testing (but at least one of stool microscopy of stool PCR performed). Active infection = Positive stool microscopy or stool PCR for S. stercoralis, regardless of other testing performed. CSF=Cerebrospinal fluid. HIV=Human immunodeficiency virus. IQR=Interquartile range. TBM=Tuberculous meningitis. WBC=White blood cell.
