# Supplementary material for: Influence of Strongyloides stercoralis Coinfection on the Presentation, Pathogenesis, and Outcome of Tuberculous Meningitis
Source: J Infect Dis. 2020 Oct 26;225(9):1653–62. doi: 10.1093/infdis/jiaa672 (PMC9071290; doi:10.1093/infdis/jiaa672)
Supplement: jiaa672_suppl_Supplementary_Table_5 [file jiaa672_suppl_supplementary_table_5.docx]

**Supplementary table 5: Median CSF cytokine concentrations by *S. stercoralis* testing group**

| **Cytokine (pg/mL)** | ***S. stercoralis* testing** | | | **Ratio of reduction** | |
| --- | --- | --- | --- | --- | --- |
|  | **Uninfected** | **Past infection** | **Active infection** | **Uninfected/ Past infection (Ratio of reduction)** | **Uninfected/ Active infection (Ratio of reduction)** |
| TNF-α | 11.94 | 4.61 | 4.49 | 2.59 (p=0.02) | 2.66 (p=0.02) |
| IFN-ɣ | 56.03 | 13.78 | 11.37 | 4.07 (p=0.02) | 4.93 (p=0.01) |
| IL-1β | 2.11 | 2.11 | 2.11 | 1.00 (p=0.26) | 1.00 (p=0.05) |
| IL-2 | 54.48 | 28.25 | 33.08 | 1.93 (p=0.03) | 1.65 (p=0.03) |
| IL-4 | 8.91 | 5.02 | 4.14 | 1.77 (p=0.12) | 2.15 (p=0.01) |
| IL-5 | 0.37 | 0.37 | 0.02 | 1.00 (p=0.02) | 18.5 (p=0.55) |
| IL-6 | 655.56 | 55.41 | 12.22 | 11.83 (p=0.10) | 53.65 (p=0.01) |
| IL-10 | 12.03 | 10.00 | 6.53 | 1.20 (p=0.17) | 1.84 (p=0.004) |
| IL-12p70 | 4.34 | 4.34 | 1.13 | 1.00 (p=0.92) | 3.84 (p=0.21) |
| IL-13 | 23.87 | 7.46 | 19.46 | 3.20 (p=0.03) | 1.23 (p=0.30) |

Median values are shown, with ratio of reduction of median values by *S. stercoralis* infection group. Cytokine concentrations are shown in pg/mL without log2 transformation. CSF=cerebrospinal fluid. IFN=interferon. IL=interleukin. TNF=tumour necrosis factor.
