# Supplementary material for: Influence of Strongyloides stercoralis Coinfection on the Presentation, Pathogenesis, and Outcome of Tuberculous Meningitis
Source: J Infect Dis. 2020 Oct 26;225(9):1653–62. doi: 10.1093/infdis/jiaa672 (PMC9071290; doi:10.1093/infdis/jiaa672)
Supplement: jiaa672_suppl_Supplementary_Table_6 [file jiaa672_suppl_supplementary_table_6.docx]

**Supplementary table 6: A restricted analysis of median CSF cytokine concentrations by *S. stercoralis* testing group, inclusive only of HIV uninfected cases, or HIV co-infected cases**

| **HIV uninfected** | | | | | |
| --- | --- | --- | --- | --- | --- |
| **Cytokine (pg/mL)** | ***S. stercoralis* testing** | | | **Ratio of reduction** | |
|  | **Uninfected**  **N=69** | **Past infection**  **N=22** | **Active infection**  **N=17** | **Uninfected/ Past infection (Wilcoxon p value)** | **Uninfected/ Active infection (Wilcoxon p value)** |
| TNF-α | 8.77 | 5.48 | 9.34 | 0.12 | 0.43 |
| IFN-ɣ | 50.39 | 17.32 | 29.91 | 0.11 | 0.17 |
| IL-1β | 2.11 | 2.11 | 2.11 | 0.60 | 0.53 |
| IL-2 | 52.61 | 28.25 | 37.12 | 0.10 | 0.21 |
| IL-4 | 8.42 | 5.75 | 5.75 | 0.16 | 0.24 |
| IL-5 | 0.37 | 0.37 | 0.37 | 0.36 | 0.54 |
| IL-6 | 289.09 | 77.62 | 56.41 | 0.25 | 0.18 |
| IL-10 | 13.41 | 11.01 | 9.67 | 0.51 | 0.10 |
| IL-12p70 | 4.34 | 4.34 | 4.34 | 0.70 | 0.77 |
| IL-13 | 19.46 | 2.88 | 19.46 | 0.08 | 0.64 |
| **HIV co-infected** | | | | | |
| **Log2 Cytokine** | ***S. stercoralis* testing** | | | **Ratio of reduction** | |
|  | **Uninfected**  **N=36** | **Past infection**  **N=4** | **Active infection**  **N=8** | **Uninfected/ Past infection (Wilcoxon p value)** | **Uninfected/ Active infection (Wilcoxon p value)** |
| TNF-α | 17.63 | 1.56 | 1.75 | 0.02 | 0.003 |
| IFN-ɣ | 61.24 | 5.26 | 5.54 | 0.03 | 0.01 |
| IL-1β | 6.09 | 0.02 | 0.02 | 0.19 | 0.04 |
| IL-2 | 69.32 | 24.16 | 17.11 | 0.29 | 0.05 |
| IL-4 | 11.17 | 4.14 | 1.41 | 0.62 | 0.02 |
| IL-5 | 0.02 | 0.33 | 0.02 | 0.10 | 0.83 |
| IL-6 | 655.56 | 23.16 | 3.95 | 0.15 | 0.01 |
| IL-10 | 11.66 | 4.48 | 3.17 | 0.02 | 0.005 |
| IL-12p70 | 1.13 | 1.13 | 1.13 | 0.69 | 0.07 |
| IL-13 | 34.79 | 23.37 | 19.46 | 0.51 | 0.11 |

P values are shown for comparison with negative group in each case. The Wilcoxon rank sum test was used to compare cytokine data. Cytokine concentrations are shown in pg/mL.
