# Supplementary material for: Influence of Strongyloides stercoralis Coinfection on the Presentation, Pathogenesis, and Outcome of Tuberculous Meningitis
Source: J Infect Dis. 2020 Oct 26;225(9):1653–62. doi: 10.1093/infdis/jiaa672 (PMC9071290; doi:10.1093/infdis/jiaa672)
Supplement: jiaa672_suppl_Supplementary_Table_7 [file jiaa672_suppl_supplementary_table_7.docx]

**Supplementary table 7: Neurological complications in *S. stercoralis* uninfected, past infection, and active infection groups**

|  | ***S. stercoralis* testing** | | |
| --- | --- | --- | --- |
|  | **Uninfected**  (N=110) | **Past infection**  (N=30) | **Active infection**  (N=26) |
| Neurological events  (No. [%]) | 33  (30.0%) | 5  (16.7%) | 1  (3.8%) |
| Fall in GCS ≥ 2 points for ≥ 48 hours  (No. [%]) | 26  (23.6%) | 4  (13.3%) | 0  (3.8%) |
| Focal neurological sign  (No. [%]) | 5  (4.5%) | 0  (0%) | 0  (0%) |
| Seizure  (No. [%]) | 2  (1.8%) | 0  (0%) | 0  (0%) |
| Paraplegia/paraparesis  (No. [%]) | 0  (0%) | 1  (3.3%) | 0  (0%) |

N = Number of participants. GCS=Glasgow coma score
