# Supplementary material for: Influence of Strongyloides stercoralis Coinfection on the Presentation, Pathogenesis, and Outcome of Tuberculous Meningitis
Source: J Infect Dis. 2020 Oct 26;225(9):1653–62. doi: 10.1093/infdis/jiaa672 (PMC9071290; doi:10.1093/infdis/jiaa672)
Supplement: jiaa672_suppl_Supplementary_Table_8 [file jiaa672_suppl_supplementary_table_8.docx]

**Supplementary table 8: Multivariate analysis of factors predicting neurological complications by 3 months**

| **Variable** | **Odds Ratio** | **95% CI** | **P value** |
| --- | --- | --- | --- |
| Age (years) | 0.99 | 0.95-1.02 | 0.38 |
| MRC TBM Grade 2 | 9.72 | 3.06-38.7 | <0.001 |
| MRC TBM Grade 3 | 11.2 | 3.05-49.1 | <0.001 |
| HIV co-infection | 2.38 | 0.84-7.03 | 0.10 |
| Active *S. stercoralis* co-infection | 0.09 | 0.00-0.54 | 0.01 |

Odds ratios with CIs are shown for the prediction of neurological complications with each variable. Age represents an increase in 1 year. MRC TBM Grade 2 and 3 represents comparisons with MRC TBM Grade 1 in each case. HIV co-infection represents comparison with HIV uninfected participants. Active *S. stercoralis* co-infection represents comparison with *S. stercoralis* uninfected participants.

Q=quartile. CI=confidence interval. HIV=human immunodeficiency virus. MRC=Medical Research Council. TBM=tuberculous meningitis.
