# Supplementary material for: Influence of Strongyloides stercoralis Coinfection on the Presentation, Pathogenesis, and Outcome of Tuberculous Meningitis
Source: J Infect Dis. 2020 Oct 26;225(9):1653–62. doi: 10.1093/infdis/jiaa672 (PMC9071290; doi:10.1093/infdis/jiaa672)
Supplement: jiaa672_suppl_Supplementary_Table_9 [file jiaa672_suppl_supplementary_table_9.docx]

**Supplementary table 9: Subgroup analyses of primary *S. stercoralis* analysis populations by HIV co-infection status**

| **HIV uninfected** | | | | | |
| --- | --- | --- | --- | --- | --- |
|  | ***S. stercoralis* testing** | | | | |
|  | **Uninfected** | **Past infection** | | **Active infection** | |
|  |  |  | P value |  | P value |
| Patients (No.) | 73 | 26 |  | 17 |  |
| Neurological complications by 3 months   - Yes (%) - No (%) | 18 (30.2 %)  55 (69.8%) | 4 (13.0%)  22 (87.0%) | 0.48 | 0 (4.8%)  17 (95.2%) | 0.03 |
| **HIV co-infected** | | | | | |
|  | ***S. stercoralis* testing** | | | | |
|  | **Uninfected** | **Past infection** | | **Active infection** | |
|  |  |  | P value |  | P value |
| Patients (No.) | 37 | 4 |  | 9 |  |
| Neurological complications by 3 months   - Yes (%) - No (%) | 15 (40.5 %)  22 (59.5%) | 1 (25.0%)  3 (75.0%) | 0.94 | 1 (11.1%)  8 (88.9%) | 0.20 |

P values are shown for group comparison with *S. stercoralis* uninfected group in each case. The chi squared test was used to compare categorical data. Uninfected = all 3 testing methods used, and all negative. Past infection = positive *S. stercoralis* serology with no positive stool testing (but at least one of stool microscopy of stool PCR performed). Active infection = Positive stool microscopy or stool PCR for S. stercoralis, regardless of other testing performed.
