# Supplementary material for: Influence of Strongyloides stercoralis Coinfection on the Presentation, Pathogenesis, and Outcome of Tuberculous Meningitis
Source: J Infect Dis. 2020 Oct 26;225(9):1653–62. doi: 10.1093/infdis/jiaa672 (PMC9071290; doi:10.1093/infdis/jiaa672)
Supplement: jiaa672_suppl_Supplementary_Table_10 [file jiaa672_suppl_supplementary_table_10.docx]

**Supplementary table 10: A comparison of neurological complications and death by 3 months in participants who had *S. stercoralis* serology performed**

|  | **Negative *S. stercoralis* serology** | **Positive *S. stercoralis* serology** | **P value** |
| --- | --- | --- | --- |
| Patients (No.) | 606 | 53 |  |
| Neurological complications  by 3 months   - Yes (%) - No (%) | 143 (23.6%)  463 (76.4%) | 8 (15.1%)  45 (84.9%) | 0.21 |
| Death by 3 months   - Yes (%) - No (%) | 156 (25.7%)  450 (74.3%) | 8 (15.1%)  45 (84.9%) | 0.12 |

P values are shown for group comparison with the chi squared test used to compare data.
