# Supplementary material for: Influence of Strongyloides stercoralis Coinfection on the Presentation, Pathogenesis, and Outcome of Tuberculous Meningitis
Source: J Infect Dis. 2020 Oct 26;225(9):1653–62. doi: 10.1093/infdis/jiaa672 (PMC9071290; doi:10.1093/infdis/jiaa672)
Supplement: jiaa672_suppl_Supplementary_Table_11 [file jiaa672_suppl_supplementary_table_11.docx]

**Supplementary table 11: A comparison of neurological complications and death by 3 months in participants** **who had *S. stercoralis* serology and stool microscopy performed**

|  | ***S. stercoralis* testing** | | | | |
| --- | --- | --- | --- | --- | --- |
|  | **Group A** | **Group B** | | **Group C** | |
|  | **Negative for *S. stercoralis* by serology and stool microscopy** | **Positive for *S. stercoralis* by serology and negative by stool microscopy** | **P value** | **Positive for *S. stercoralis* by both serology and by stool microscopy** | **P value** |
| Patients (No.) | 475 | 37 |  | 7 |  |
| Neurological complications by 3 months   - Yes (%) - No (%) | 106 (22.3%)  369 (77.7%) | 4 (8.1%)  33 (91.9%) | 0.15 | 0  7 | 0.34 |
| Death by 3 months   - Yes (%) - No (%) | 111 (23.4%)  364 (76.6%) | 4 (10.8%)  33 (89.2%) | 0.12 | 0  7 | 0.31 |

P values are shown for comparison with negative group in each case. The chi squared test was used to compare data.
