# Supplementary material for: Influence of Strongyloides stercoralis Coinfection on the Presentation, Pathogenesis, and Outcome of Tuberculous Meningitis
Source: J Infect Dis. 2020 Oct 26;225(9):1653–62. doi: 10.1093/infdis/jiaa672 (PMC9071290; doi:10.1093/infdis/jiaa672)
Supplement: jiaa672_suppl_Supplementary_Materials_1 [file jiaa672_suppl_supplementary_materials_1.docx]

**Supplementary material 1**

Guidelines for treatment of tuberculosis [20,21]

First line treatment

Rifampicin (10mg/kg/24 hrs; maximum 600mg), isoniazid (5mg/kg/24hrs; maximum 300mg), pyrazinamide (25mg/kg/24hrs; maximum 2g) and ethambutol (20mg/kg/24 hrs; maximum 1.2g) are given for for at least the first 2 months of treatment.

Pyrazinamde will then be stopped and rifampicin, isoniazid and ethambutol (at the same doses) will then be given until at least 12 months anti-tuberculosis treatment in total has been given. If pyrazinamide cannot be given for at least 2 months (for example, because of drug-induced toxicity), then total treatment should be at least 12 months.

Isoniazid-resistant tuberculosis

Option 1: Follow the standard regimen above, but replace isoniazid with levofloxacin (20mg/kg/24 hrs; maximum 1000 mg/day). Pyrazinamide can be used throughout treatment in those with more severe disease who are responding slowly.

Option 2: Stop isoniazid and treat with rifampicin, ethambutol and pyrazinamide for the entire 9-12 months of treatment. This option is not suitable for those with confirmed ethambutol resistant bacteria; these participants should be treated with option 1.

Multi-drug resistant tuberculosis

Second line treatment for MDR TBM should be given as soon as possible, following National guidelines and local policies.
